# Effects of CPAP on Testosterone Levels in Patients With Obstructive Sleep Apnea: A Meta-Analysis Study

**DOI:** 10.3389/fendo.2019.00551

**Published:** 2019-08-21

**Authors:** Angelo Cignarelli, Marco Castellana, Giorgio Castellana, Sebastio Perrini, Francesco Brescia, Annalisa Natalicchio, Gabriella Garruti, Luigi Laviola, Onofrio Resta, Francesco Giorgino

**Affiliations:** ^1^Section of Internal Medicine, Endocrinology, Andrology and Metabolic Diseases, Department of Emergency and Organ Transplantation, University of Bari Aldo Moro, Bari, Italy; ^2^Pulmonary Division, ICS Maugeri Spa SB, IRCCS Cassano delle Murge, Cassano delle Murge, Italy; ^3^Institute of Respiratory Diseases, University of Bari “Aldo Moro”, Bari, Italy

**Keywords:** obstructive sleep apnea, continuous positive airway pressure, testosterone, hypogonadism–hypotestosteronemia–androgen deficiency, obesity complications

## Abstract

**Background:** Obstructive sleep apnea (OSA) represents a frequent complication among patients with obesity and has been associated with neuroendocrine changes, including hypogonadism.

**Objective:** We conducted a systematic review and meta-analysis to evaluate the effects of continuous positive airway pressure (CPAP) on testosterone and gonadotropins in male patients with OSA.

**Methods:** The review was registered on PROSPERO (CRD42018103164). PubMed, Scopus, CENTRAL, and Clinicaltrials.gov were searched until June 2018. Studies reporting the effect of CPAP on total testosterone, free testosterone, sexual hormone binding globulin (SHBG), follicle stimulating hormone (FSH), luteinizing hormone (LH), and prolactin were included. A subgroup analysis on hypogonadal vs. eugonadal status at baseline was performed.

**Results:** Out of 129 retrieved papers, 10 prospective cohort and 2 randomized controlled studies were included in the review. Three hundred eighty-eight patients were included. CPAP use was not associated with a significant change in total testosterone levels [mean difference 1.08, 95% confidence interval (CI) −0.48 to 2.64] or other outcomes. The subgroup analysis confirmed the overall results.

**Conclusions:** The present review does not support the hypothesis of a direct interaction between OSA and testosterone. Strategies other than CPAP should therefore be considered in managing hypogonadism in patients with OSA.

## Introduction

Obstructive sleep apnea (OSA) syndrome is a clinical condition characterized by recurrent episodes of complete or partial obstruction of the upper airway (apnea/hypopnea), leading to intermittent hypoxia, sleep fragmentation, hypercapnia, marked swing in intrathoracic pressure, and increased sympathetic activity (1). It is widely diffused in the general population, ranging from 9 to 38% (2), with a higher prevalence in men than women and in patients with obesity compared to patients with overweight, reaching a prevalence of 50–60% (2–4). Untreated OSA syndrome has been associated with cardiovascular, metabolic, and neurocognitive disorders, including hypertension, atrial fibrillation, stroke, myocardial infarction, dyslipidemia, diabetes mellitus, increased motor vehicle accidents, and decreased quality of life, causing a major economic impact on affected people, their families, and the health care system (5, 6).

Several studies have shown that sleep disorders may influence testosterone production (7–10). Current guidelines recommend distinguishing between organic and functional causes of hypogonadism. The former is characterized by a permanent dysfunction of the hypothalamus–pituitary–testicular (HPT) axis, and testosterone replacement therapy (TRT) should thus be considered in order to improve sexual function, well-being, bone mineral density, and body composition. On the other hand, the latter can be potentially reversed by treating the underlying etiology. Among the causes of functional hypogonadism, sleep disorders are listed (11). It is worth noting that the interaction between testosterone and OSA syndrome has not be fully characterized. The rise of testosterone is mostly dependent on sleep integrity, generally reaching its peak during the first 3 h of uninterrupted sleep (10); indeed, a reduced LH pulsatility and serum total testosterone has been found to be associated with sleep fragmentation and/or intermittent hypoxia occurring in patients with OSA syndrome (12, 13). However, it has been suggested that serum testosterone levels may be affected primarily by obesity in men with OSA syndrome (14, 15). Indeed, the association of testosterone levels with sleep quality and sleep-disordered breathing was absent or markedly attenuated after adjusting for body mass index (BMI) or waist circumference (16).

The first choice for treatment of moderate–severe OSA is continuous positive airway pressure (CPAP) (17). Thus, we performed a systematic review and meta-analysis to evaluate the effects of CPAP on serum testosterone and gonadotropin levels in male patients with OSA syndrome.

## Methods

The systematic review was registered in PROSPERO (registration number CRD42018103164) and performed in accordance with the Meta-analyses Of Observational Studies in Epidemiology (MOOSE) statement (Table S1).

### Search Strategy

A six-step search strategy was planned. First, we searched sentinel studies in PubMed. Second, keywords and Medical Subject Headings (MeSH) terms were identified in PubMed. Third, the terms “continuous positive airway pressure,” “testosterone,” and “gonadotropins” [including luteinizing hormone (LH), follicle stimulating hormone (FSH), and prolactin (PRL)] were searched in PubMed, in order to test the strategy. Fourth, PubMed, CENTRAL, ClinicalTrials.gov, and Scopus were searched. Fifth, observational studies and randomized clinical trials (RCTs) were selected. Sixth, references of included studies were searched for additional papers. The last search was performed on June 26, 2018. No language restriction was adopted. Two investigators (MC, AC) independently searched papers, screened titles, and abstracts of the retrieved articles, reviewed the full texts, and selected articles for their inclusion.

### Data Extraction

The following information was extracted independently by two investigators (MC, AC) in a piloted form: (1) general information on the study (author, year of publication, country, study type, number of patients, age, sex, BMI, OSA syndrome severity); (2) type of CPAP (nasal, oral) and compliance; (3) total testosterone; and (4) secondary outcomes [free testosterone, sexual hormone binding globulin [SHBG], LH, FSH, PRL]. The main paper and supplementary data were searched; if data were missing, authors were contacted *via* email. Data were cross-checked, and any discrepancy was discussed.

### Study Quality Assessment

The risk of bias of including observational studies was assessed independently by two reviewers (MC, AC) through the National Heart, Lung, and Blood Institute Quality Assessment Tool for Before–After (Pre–Post) Studies With No Control Group (18). The risk of bias of including RCTs was assessed independently by the same reviewers through the Cochrane Collaboration's tool for assessing risk of bias for the following aspects: random sequence generation, allocation concealment, blinding of participants and personnel, blinding of outcome assessment, incomplete outcome data, and selecting reporting. For other bias, funding was assessed. Each domain was assigned as low, unclear, or high risk of bias (19).

### Data Analysis

The primary outcome was the change in serum total testosterone from baseline to the last available follow-up. Secondary outcomes included change in serum free testosterone, SHBG, LH, FSH, and PRL from baseline to the last available follow-up. The end points were analyzed as continuous variables and summarized as weighted-mean difference. If standard deviation was missing in a study for a specific outcome, it was calculated from standard error, 95% confidence interval (CI), or interquartile range; if none of these were available, the largest among the other studies was reported. In order to assess differences between eugonadal and hypogonadal subjects, a subgroup analysis was planned with a cutoff for serum total testosterone of 12 nmol/l. Heterogeneity between studies was assessed by using *I*^2^, with 50% or higher regarded as high. Publication bias was assessed with Egger's test and funnel plot visually; the trim-and-fill method was used for estimating its effect. Sensitivity analyses by removing each study in turn were also performed. All analyses were two-sided and were carried out using RevMan 5.3 (Cochrane Collaboration) and RStudio ver. 1.1.383 (RStudio Team) with a random-effect model; *p* < 0.05 was regarded as significant.

## Results

### Study Characteristics

**Figure 1:**
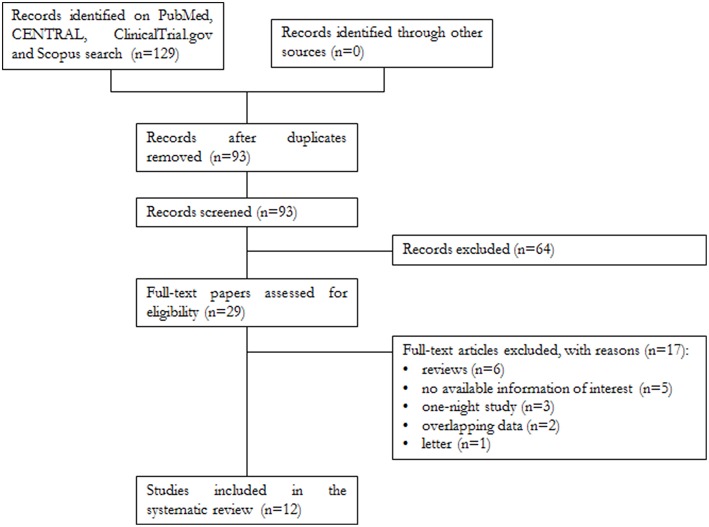
Flowchart of the systematic review.

A total of 129 papers were found, of which 32 were on PubMed, 58 on Scopus, 38 on CENTRAL, and 1 on ClinicalTrials.gov. After removal of 36 duplicates, 93 articles were analyzed for title and abstract; 64 records were excluded (systematic reviews, meta-analyses, case reports, articles not in the field of the review). The remaining 29 papers were retrieved in full text, and 12 articles were finally included in the systematic review (Figure 1).

### Study Quality Assessment

The risk of bias of the included studies is shown in supplementary data (Table S2, Figures S1–S3). Concerning the observational studies, statement of the study question, eligibility criteria, representativeness and enrollment of patients, and statistical analysis were adequate in all. Sample size calculation was reported in two papers (20, 21) and adherence to CPAP in five (20–24). Only in Knapp et al. serum testosterone was measured by liquid chromatography mass spectrometry, which is considered as the most reliable method (22, 25). The loss to follow-up was 20% or less in five studies (9, 23, 24, 26). The outcome measures of interest were taken more than once after CPAP in two studies (22, 27).

Concerning the two RCTs, data on allocation concealment were not reported; the remaining domains were adequate in both (28, 29).

### Qualitative Analysis (Systematic Review)

The characteristics of the included articles are summarized in Table 1. The studies were published between 1989 and 2017 and had sample sizes ranging from 5 to 101 patients and a follow-up from 4 to 156 weeks. Ten studies were prospective cohort and two randomized controlled. Eight studies examined oral CPAP and four nasal CPAP. Seven studies reported data on compliance to CPAP. Participants were adult outpatients diagnosed with OSA syndrome; five trials enrolled patients with serum total testosterone <12 nmol/l at baseline. The weighted-mean age was 52.6 ± 11.3 years, and the weighted-mean BMI was 32.5 ± 5.5 kg/m^2^. A total of 388 male patients were included, of which 245 received oral and 143 nasal CPAP; information on the presence of diabetes was available for 44 patients (11%) (22, 29). Moreover, 49 patients were treated with sham-CPAP, 21 with mandibular advancement devices, and 12 with CPAP and simultaneous TRT; they were not included in the present review.

**Table 1:** Qualitative analysis of studies included in the systematic review.

**References**	**CPAP**	**Comparator**	**Study type**	**Follow-up (weeks)**	**Number of patients in CPAP arm**	**Population**	**Age (years)**	**BMI (kg/m^**2**^)**	**Total testosterone**	**Free testosterone**	**SHGB**	**FSH**	**LH**	**PRL**
**TOTAL TESTOSTERONE** **<** **12 NMOL/L AL BASELINE**														
Li et al. (20)	Nasal CPAP		PCS	4	32	Severe OSA and erectile dysfunction	55.5	28.5	x			x	x	x
Luboshitzky et al. (9)	Oral CPAP		PCS	36	5	Severe OSA	49.5	31.7	x				x	
Macrea et al. (21)	Oral CPAP		PCS	44–156	10	Moderate OSA	57	32	x			x	x	x
Madaeva et al. (30)	Oral CPAP	CPAP + AndroGel	PCS	8	14	Moderate OSA and hypogonadism	46.1	35.2	x					
Madaeva et al. (26)	Oral CPAP		PCS	8	34	Moderate OSA	60.5	33.4	x					
**TOTAL TESTOSTERONE** **≥** **12 NMOL/L AL BASELINE**														
Bratel et al. (31)	Nasal CPAP		PCS	28	16	Moderate–severe OSA	51.3	32	x	x	x		x	x
Celec et al. (27)	Oral CPAP		PCS	24	67	Severe OSA	56.2	33.7	x					
Grunstein et al. (23)	Nasal CPAP		PCS	12	43	Severe OSA	53	33	x	x	x			
Hoekema et al. (28)	Oral CPAP	Oral appliance	RCT	8–12	27	Non-severe–severe OSA	49.9	31	x	x				
Knapp et al. (22)	Oral CPAP		PCS	12	35	OSA and type 2 diabetes mellitus	65.4	32	x	x	x			
Meston et al. (29)	Nasal CPAP	Sham-CPAP	RCT	4	52	Moderate–severe OSA	49	35	x		x	x	x	x
Zhang et al. (24)	Oral CPAP		PCS	12	53	Severe OSA	44.6	28.3	x			x	x	x

*BMI, body mass index; CPAP, continuous positive airway pressure; FSH, follicle stimulating hormone; LH, luteinizing hormone; OSA, obstructive sleep apnea; PCS, prospective cohort study; PRL, prolactin; RCT, randomized clinical trial; SHBG, sex hormone binding globulin. “x,” retrieved data*.

### Quantitative Analysis (Meta-Analysis)

**Figure 2:**
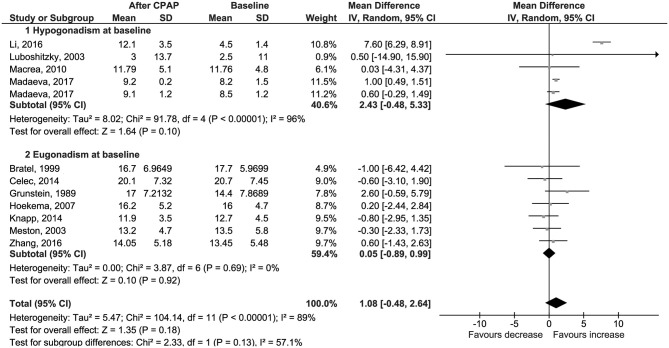
Forest plots of meta-analysis for change in total testosterone.

The primary outcome was the change in serum total testosterone levels from baseline to the last available follow-up. Data were available for 95 patients with hypogonadism and 280 with eugonadism at baseline. The weighted-mean serum total testosterone at baseline was 13.5 ± 7.4 nmol/l, and it was different between the two groups (6.8 ± 3.5 nmol/l in hypogonadal and 15.7 ± 7.0 nmol/l in eugonadal patients). CPAP use was not associated with a change in serum total testosterone levels (Δ = 1.08 nmol/l, 95% CI −0.48 to 2.64, *p* = 0.18, *I*^2^ = 89%); results were confirmed by the subgroup analysis in eugonadal and hypogonadal men, even though a trend toward increased serum testosterone was noted in the latter subgroup (Figure 2). Sensitivity analysis had not reached statistical significance (Table S3); a funnel plot showed that a possible publication bias may exist, although Egger's test was not statistically significant (*p* = 0.356; Figure S4).

**Figure 3:**
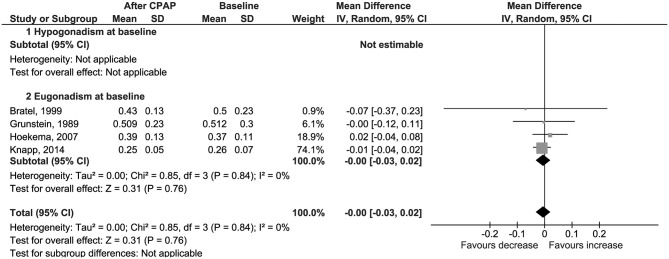
Forest plots of meta-analysis for change in free testosterone.

**Figure 4:**
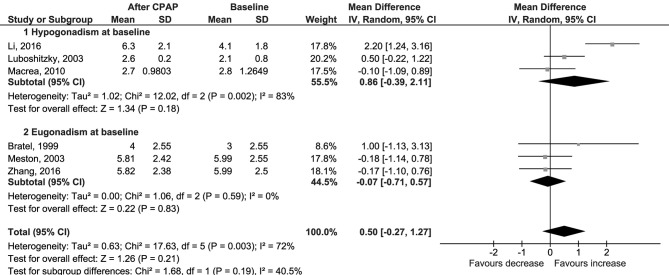
Forest plots of meta-analysis for change in LH.

**Figure 5:**
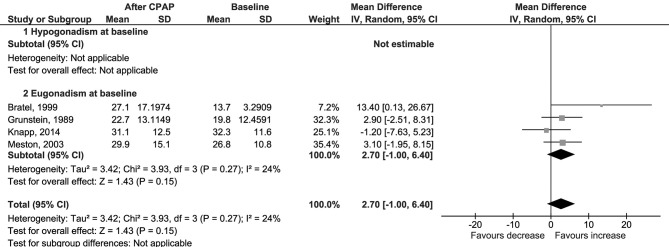
Forest plots of meta-analysis for change in SHBG.

No difference was found also for changes in levels of serum free testosterone (Δ = 0.00 nmol/l, 95% CI −0.03 to 0.02, *p* = 0.77, *I*^2^ = 0%; Figure 3); LH (Δ = 0.50 IU/l, 95% CI −0.27 to 1.27, *p* = 0.21, *I*^2^ = 72%; Figure 4); FSH (Δ = 1.97 IU/l, 95% CI −1.54 to 5.48, *p* = 0.27, *I*^2^ = 96%; Figure S5); PRL (Δ = −0.52 μg/l, 95% CI −1.86 to 0.81, *p* = 0.44, *I*^2^ = 76%; Figure S6); and SHBG (Δ = 2.70 nmol/l, 95% CI −1.00 to 6.40, *p* = 0.15, *I*^2^ = 24%; Figure 5).

## Discussion

The aim of this systematic review and meta-analysis was to identify the best available evidence on the efficacy of CPAP on serum total testosterone levels in male patients with OSA syndrome. Twelve studies were found including adult patients with eugonadism or hypogonadism at baseline. The overall results of our meta-analysis showed that CPAP does not influence total testosterone or gonadotropins.

To our knowledge, this is the first systematic review and meta-analysis assessing differences due to CPAP use in eugonadal and hypogonadal patients with OSA syndrome, as well as focusing on gonadotropins. Papers were searched without time restrictions, inclusion criteria were defined prior to the database search, and data were searched on original articles and supplementary data.

Two hypotheses have been formulated in order to describe the interactions between OSA syndrome and serum testosterone. According to some authors, sleep fragmentation and/or intermittent hypoxia occurring in untreated OSA syndrome may influence the HPT axis, reducing Gonadotropin Releasing Hormone (GnRH) secretion, LH pulsatility, and serum total testosterone (12, 13). The mechanism of this interaction has not been fully characterized, but it could probably be mediated by endorphins (12, 32, 33). An adequate treatment with CPAP could be sufficient for the correction of hypogonadism, indeed (20). Conversely, according to other authors, overweight and obesity represent the “common soil” for both OSA syndrome and hypogonadism. Overweight and obesity are regarded as a major risk factor for OSA syndrome, acting through both mechanical and biochemical mechanisms, such as changes in peri-pharyngeal soft tissues and depression of neuromuscular control (34). On the other the hand, visceral adiposity is associated with impaired synthesis of GnRH in the hypothalamus, SHBG in the liver, and testosterone in Leydig cells (35–38). In supporting this link, body weight loss promotes a decrease in apnea–hypopnea index as well as an increase in serum total and free testosterone levels (39, 40). However, since CPAP *per se* does not lead to a decrease in body weight, no effects on the HPT axis should be expected (41, 42). The present meta-analysis supports this hypothesis rather than a direct link between OSA syndrome and hypogonadism. Strategies other than CPAP should thus be considered in these patients, including weight loss, exercise, and optimization of concomitant chronic diseases to improve gonadal function (43).

In male patients with obesity, low levels of SHBG are consistently reported. Since ~70% of serum testosterone is bound to SHBG, current guidelines recommend determining the androgen status through the evaluation of serum free testosterone, either directly from equilibrium dialysis assays or by calculations that use serum total testosterone, SHBG, and albumin (11). In the present review, no data on serum free testosterone or SHBG in patients with hypogonadism were available. It is worth noting that other treatments, such as diet and bariatric surgery, proved to increase total as well as serum free testosterone and SHBG (40). Since CPAP has shown a neutral effect on serum total testosterone, no different result should be expected for the other outcomes, indeed (43).

Eighty-two patients were excluded from the present review: 49 were treated with sham-CPAP, 21 with mandibular advancement devices, and 12 with CPAP and simultaneous TRT. A significant increase in serum total testosterone was reported only in the third group (28–30). It is worth mentioning that untreated severe OSA syndrome contraindicates TRT, due to a time-limited worsening of the sleep disorder (11, 44).

In December 2014, a meta-analysis on the same topic was published by Zhang et al. The authors stated that CPAP does not influence serum total testosterone, free testosterone, and SHBG. These results were drawn from the analysis of seven studies, all included in the present paper (9, 22, 23, 27–29, 31). Of note, only one of them enrolled patients with low serum testosterone (24). Our meta-analysis is in line with previous work, and conclusions on hypogonadal patients are strengthened by the subgroup analysis.

This review has several limitations. The first limitation relates to the design of the included papers: the majority was represented by observational studies with low number of participants and short duration. Secondly, the efficacy of CPAP strictly depends on adherence to the treatment and correction of apnea/hypopnea as well as nocturnal hypoxia. In particular, lack of compliance is regarded as a major issue in OSA syndrome management. In the present systematic review, only three studies reported an adequate CPAP use (4 h per night on at least 70% of nights) (20, 21, 28, 45), and thus, results might reflect, at least in part, suboptimal CPAP therapy. This threshold, although defined as arbitrary, has proved useful in clinical studies and has some validity. Indeed, adherence to CPAP therapy for at least 4 h per night has been associated with normalization of daytime sleepiness, improvement of quality of life and neurocognitive function (46–49), and improvements in cardiovascular disease conditions and diabetes (50–53). Also, CPAP adherence is usually defined as hours per night rather than as a proportion of total sleep time, which is usually not measured. So far, it is unclear if shorter sleep duration with high CPAP adherence would be associated with better outcomes than longer sleep duration with low CPAP adherence. Concerning the latter aspect, it is estimated that CPAP is not able to correct all the nocturnal events in about 20% of patients with OSA syndrome; the risk is increased in patients with concomitant obesity hypoventilation syndrome and chronic obstructive pulmonary disease (54). Among the included studies, only six reported the correction of apnea/hypopnea (20, 21, 26, 28, 30, 31) and three the resolution of nocturnal hypoxia (9, 26, 30). A high heterogeneity for four out of six evaluated outcomes was found, and this is a third limitation. This could be due to: (1) study design and (2) patient characteristics other than the extracted ones. In particular, the data in Li et al. (20) are not in line with the other studies, although no clear reason could be found in the study protocol. Caution should thus be taken in generalizing results to clinical practice. Lastly, it should be considered that pituitary imaging in patients with serum total testosterone <5.2 nmol/l and low or inappropriately normal gonadotropins is recommended in order to exclude secondary organic causes (11). Two studies included such patients, but no information other than a “medical history” evaluation is reported (9, 20). In all, a limited number of studies specifically focusing on the effect of CPAP in hypogonadal patients was found, and this prevented any additional analysis (e.g., meta-regression) to explore the high heterogeneity of findings. Further studies are thus needed reporting data on sleep duration, compliance to CPAP, months of use, OSA syndrome improvement, and BMI.

## Conclusions

In male patients with OSA syndrome, CPAP use is associated with a neutral effect on serum total testosterone and gonadotropins, regardless of the gonadal status at baseline. In managing hypogonadism in patients with OSA syndrome, strategies other than CPAP should therefore be considered.

## Data Availability

The datasets generated during and/or analyzed during the current study are not publicly available but are available from the corresponding author on reasonable request.

## Author Contributions

MC, AC, and GC conceived the meta-analysis, developed the search strategy, provided statistical expertise, and drafted the manuscript. All authors contributed to the development of the selection criteria, the risk-of-bias assessment strategy, and data extraction criteria. All authors read, provided feedback, and approved the final manuscript.

### Conflict of Interest Statement

The authors declare that the research was conducted in the absence of any commercial or financial relationships that could be construed as a potential conflict of interest.
