# Supplementary material for: Effects of CPAP on Testosterone Levels in Patients With Obstructive Sleep Apnea: A Meta-Analysis Study
Source: Front Endocrinol (Lausanne). 2019 Aug 21;10:551. doi: 10.3389/fendo.2019.00551 (PMC6712440; doi:10.3389/fendo.2019.00551)

Supplementary Figure 1. Risk of bias graph: review of authors' judgements about each risk of bias item presented as percentages across all included observational studies.

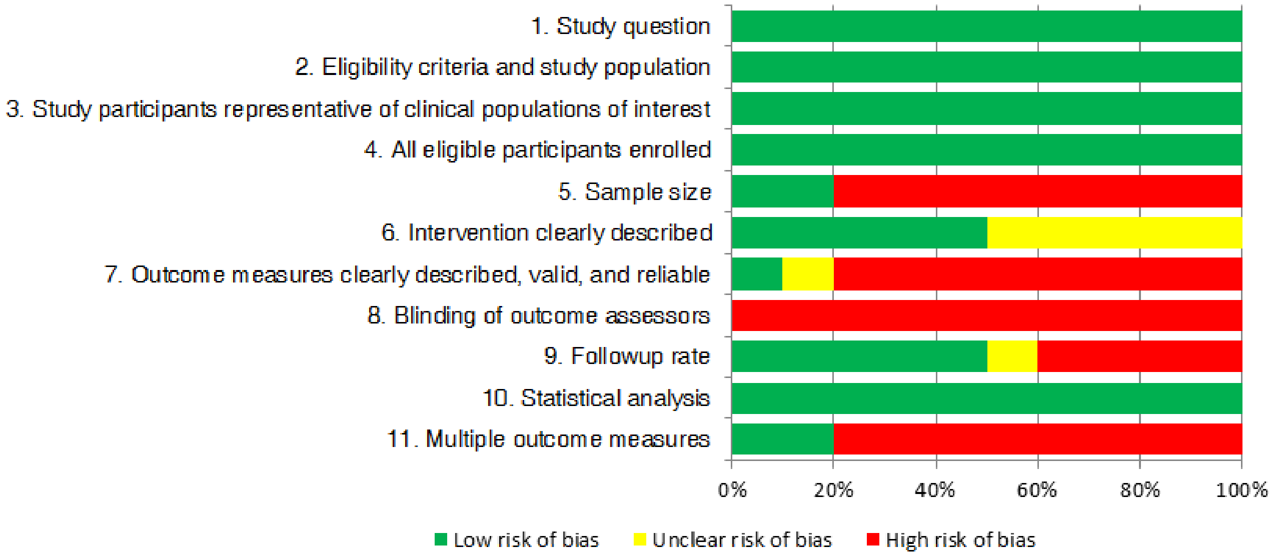

Supplement: Supplementary file 1 [file Image_1.pdf]
