# Supplementary figures and images for: Effects of CPAP on Testosterone Levels in Patients With Obstructive Sleep Apnea: A Meta-Analysis Study
Source: Front Endocrinol (Lausanne). 2019 Aug 21;10:551. doi: 10.3389/fendo.2019.00551 (PMC6712440; doi:10.3389/fendo.2019.00551)

**Supplementary Figure 4.** Funnel plot of meta-analysis for change in total testosterone.

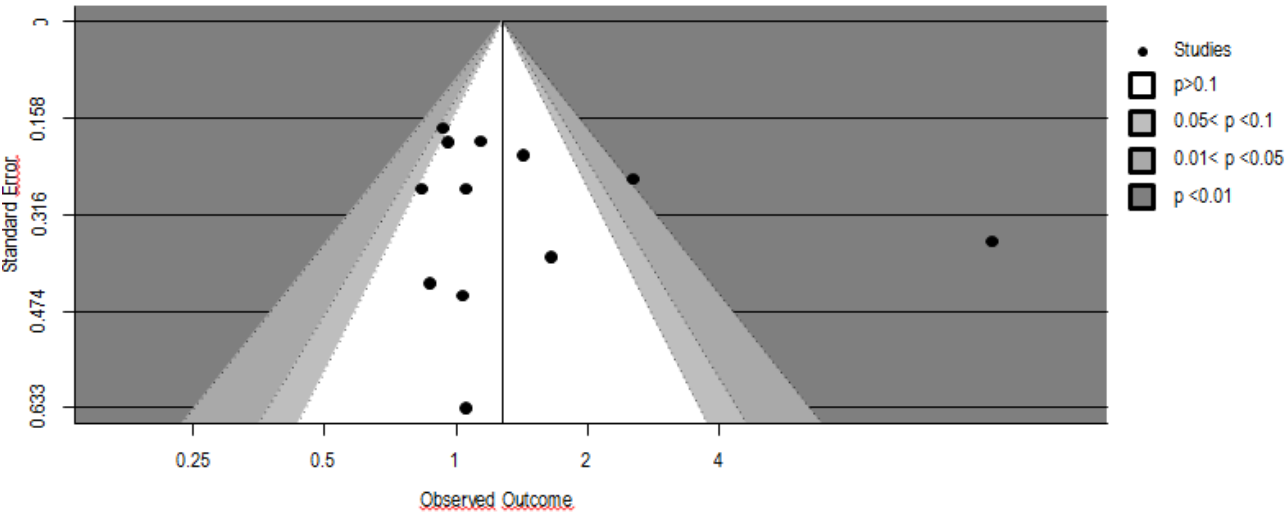

Supplement: Supplementary file 4 [file Image_4.pdf]

**Supplementary Figure 5.** Forest plots of meta-analysis for change in FSH.

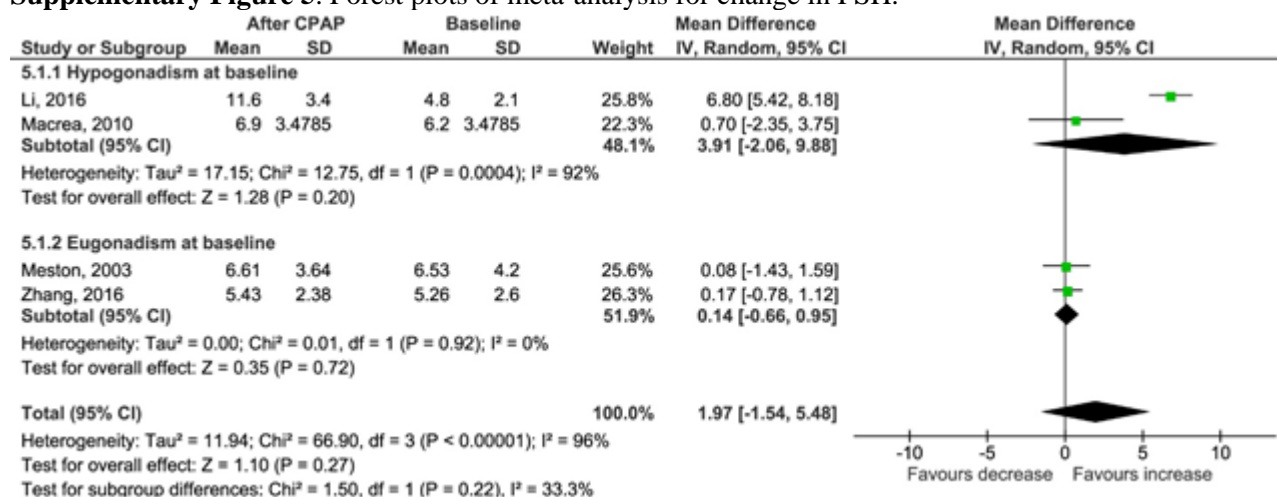

Supplement: Supplementary file 5 [file Image_5.pdf]

**Supplementary Figure 6.** Forest plots of meta-analysis for change in PRL.

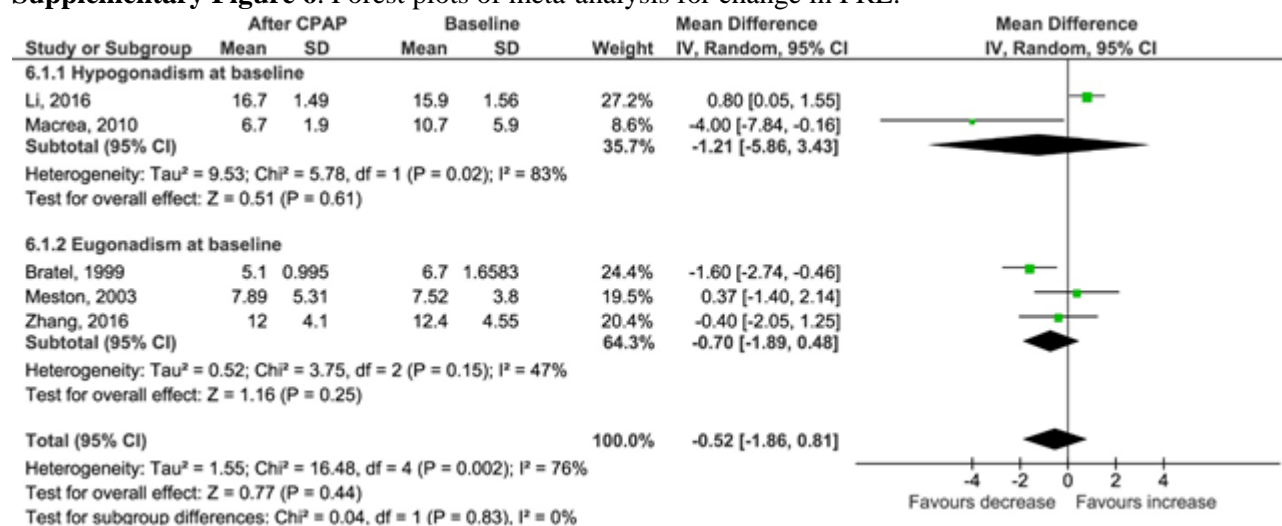

Supplement: Supplementary file 6 [file Image_6.pdf]
