# Supplementary material for: Effects of CPAP on Testosterone Levels in Patients With Obstructive Sleep Apnea: A Meta-Analysis Study
Source: Front Endocrinol (Lausanne). 2019 Aug 21;10:551. doi: 10.3389/fendo.2019.00551 (PMC6712440; doi:10.3389/fendo.2019.00551)
Supplement: Supplementary file 8 [file Table_2.docx]

Effects of CPAP on testosterone levels in patients with obstructive sleep apnea: a meta-analysis study.

Angelo Cignarelli^1^, Marco Castellana^1^, Giorgio Castellana^2^, Sebastio Perrini^1^, Francesco Brescia^1^, Annalisa Natalicchio^1^, Gabriella Garruti^1^, Luigi Laviola^1^, Onofrio Resta^3^, Francesco Giorgino^1,*^

^1^ Section of Internal Medicine, Endocrinology, Andrology and Metabolic Diseases, Department of Emergency and Organ Transplantation, University of Bari “Aldo Moro”, Bari, Italy.

^2^ Pulmonary Division, ICS Maugeri Spa SB, IRCCS Cassano delle Murge, Italy

^3^ Institute of Respiratory Diseases, University of Bari “Aldo Moro”, Bari, Italy.

* Corresponding author: Francesco Giorgino, Section of Internal Medicine, Endocrinology, Andrology and Metabolic Diseases, Department of Emergency and Organ Transplantation, University of Bari Aldo Moro, Bari, Italy. Tel. +39 0805593522, Fax +39 0805478151, E-mail [francesco.giorgino@uniba.it](mailto:francesco.giorgino@uniba.it)

**Table S2.** Risk of bias summary: review of authors’ judgements about each risk of bias item for each included observational study.

|  | **1** | **2** | **3** | **4** | **5** | **6** | **7** | **8** | **9** | **10** | **11** | **Total** |
| --- | --- | --- | --- | --- | --- | --- | --- | --- | --- | --- | --- | --- |
| Bratel, 1999 (37) | Yes | Yes | Yes | Yes | No | NR | No | No | No | Yes | No | 5/11 |
| Celec, 2014 (20) | Yes | Yes | Yes | Yes | No | NR | No | No | NR | Yes | Yes | 6/11 |
| Grunstein, 1989 (16) | Yes | Yes | Yes | Yes | No | Yes | No | No | Yes | Yes | No | 7/11 |
| Knapp, 2014 (15) | Yes | Yes | Yes | Yes | No | Yes | Yes | No | No | Yes | Yes | 8/11 |
| Li, 2016 (13) | Yes | Yes | Yes | Yes | Yes | Yes | No | No | Yes | Yes | No | 8/11 |
| Luboshitzky, 2003 (24) | Yes | Yes | Yes | Yes | No | NR | No | No | Yes | Yes | No | 6/11 |
| Macrea, 2010 (14) | Yes | Yes | Yes | Yes | Yes | Yes | No | No | No | Yes | No | 7/11 |
| Madaeva, 2017 (35) | Yes | Yes | Yes | Yes | No | NR | No | No | Yes | Yes | No | 6/11 |
| Madaeva, 2017 (19) | Yes | Yes | Yes | Yes | No | NR | No | No | No | Yes | No | 5/11 |
| Zhang, 2016 (17) | Yes | Yes | Yes | Yes | No | Yes | NR | No | Yes | Yes | No | 7/11 |

Questions:

1. Was the study question or objective clearly stated?

2. Were eligibility/selection criteria for the study population prespecified and clearly described?

3. Were the participants in the study representative of those who would be eligible for the test/service/intervention in the general or clinical population of interest?

4. Were all eligible participants that met the prespecified entry criteria enrolled?

5. Was the sample size sufficiently large to provide confidence in the findings?

6. Was the test/service/intervention clearly described and delivered consistently across the study population?

7. Were the outcome measures prespecified, clearly defined, valid, reliable, and assessed consistently across all study participants?

8. Were the people assessing the outcomes blinded to the participants' exposures/interventions?

9. Was the loss to follow-up after baseline 20% or less? Were those lost to follow-up accounted for in the analysis?

10. Did the statistical methods examine changes in outcome measures from before to after the intervention? Were statistical tests done that provided p values for the pre-to-post changes?

11. Were outcome measures of interest taken multiple times before the intervention and multiple times after the intervention (i.e., did they use an interrupted time-series design)?
