# Supplementary material for: Effects of CPAP on Testosterone Levels in Patients With Obstructive Sleep Apnea: A Meta-Analysis Study
Source: Front Endocrinol (Lausanne). 2019 Aug 21;10:551. doi: 10.3389/fendo.2019.00551 (PMC6712440; doi:10.3389/fendo.2019.00551)
Supplement: Supplementary file 9 [file Table_3.docx]

Effects of CPAP on testosterone levels in patients with obstructive sleep apnea: a meta-analysis study.

Angelo Cignarelli^1^, Marco Castellana^1^, Giorgio Castellana^2^, Sebastio Perrini^1^, Francesco Brescia^1^, Annalisa Natalicchio^1^, Gabriella Garruti^1^, Luigi Laviola^1^, Onofrio Resta^3^, Francesco Giorgino^1,*^

^1^ Section of Internal Medicine, Endocrinology, Andrology and Metabolic Diseases, Department of Emergency and Organ Transplantation, University of Bari “Aldo Moro”, Bari, Italy.

^2^ Pulmonary Division, ICS Maugeri Spa SB, IRCCS Cassano delle Murge, Italy

^3^ Institute of Respiratory Diseases, University of Bari “Aldo Moro”, Bari, Italy.

* Corresponding author: Francesco Giorgino, Section of Internal Medicine, Endocrinology, Andrology and Metabolic Diseases, Department of Emergency and Organ Transplantation, University of Bari Aldo Moro, Bari, Italy. Tel. +39 0805593522, Fax +39 0805478151, E-mail [francesco.giorgino@uniba.it](mailto:francesco.giorgino@uniba.it)

**Table S3**. Sensitivity analysis for change in total testosterone.

| Removed study | Estimate | (95% Confidence Interval) | p-value |
| --- | --- | --- | --- |
| Li, 2016 (13) | 0.1382 | (-0.0631; 0.3396) | 0.1785 |
| Luboshitzky, 2003 (24) | (0.3702 | (-0.0378; 0.7782) | 0.0754 |
| Macrea, 2010 (14) | 0.378 | (-0.0365; 0.7925) | 0.0739 |
| Madaeva, 2017 (35) | 0.3421 | (-0.0756; 0.7597) | 0.1084 |
| Madaeva, 2017 (19) | 0.2955 | (-0.1130; 0.7040) | 0.1563 |
| Bratel, 1999 (37) | 0.3918 | (-0.0219; 0.8056) | 0.0635 |
| Celec, 2014 (20) | 0.4004 | (-0.0398; 0.8407) | 0.0746 |
| Grunstein, 1989 (16) | 0.3551 | (-0.0841; 0.7942) | 0.113 |
| Hoekema, 2007 (21) | 0.3839 | (-0.0440; 0.8118) | 0.0787 |
| Knapp, 2014 (15) | 0.4063 | (-0.0150; 0.8276) | 0.0587 |
| Meston, 2003 (22) | 0.3969 | (-0.0405; 0.8342) | 0.0753 |
| Zhang, 2016 (17) | 0.3786 | (-0.0663; 0.8235) | 0.0953 |
